# INTEREST GROUP SESSION—GEROSCIENCE: METHODS FROM BENCH TO POPULATION SCIENCE TO INFORM CONSTRUCTION OF GEROSCIENCE CLINICAL TRIALS

**DOI:** 10.1093/geroni/igz038.2728

**Published:** 2019-11-08

**Authors:** Jason L Sanders, Anne B Newman

**Affiliations:** 1 Brigham and Women’s Hospital, Boston, Massachusetts, United States; 2 University of Pittsburgh, Pittsburgh, Pennsylvania, United States

## Abstract

We are on the cusp of a revolution in aging science. It has matured to the point where geroscience trials will test interventions in humans which alter aging mechanisms to lengthen healthspan and possibly lifespan. This goal is unprecedented in clinical trial design, and it requires retooling the clinical trial toolbox. Traditionally, trials are constructed around a single disease; interventions target a narrow part of a defined biological pathway involving only one molecule, tissue, or organ; events are well known intermediate endpoints and clinically-defined hard outcomes; and follow up may be short and historically informed based on prior trials. Geroscience trials by design target aging mechanisms which, when altered, are likely to have pleiotropic effects that modify several biologic pathways; efficacy and safety signals may require integration across multiple levels of biologic organization; intermediate endpoints are not agreed upon; and follow up timelines are undefined. In this symposium, we provide guidance on the design of geroscience trials using examples that span from bench to population science. Dr. LeBrasseur will discuss screening senolytic compounds across models of age-associated decline and advancing their candidacy as interventions. Dr. Justice will detail a framework for biomarker selection in geroscience trials, focusing on a trial of metformin as an example. Dr. Sanders will illustrate how observational data can inform phenotype use in clinical trials. Dr. Levine will explain translating omics data for use in geroscience trials, focusing on epigenomics. We expect additional discussion to hasten development of well-designed geroscience trials.

